# Performance Enhancement of a GaAs Detector with a Vertical Field and an Embedded Thin Low-Temperature Grown Layer

**DOI:** 10.3390/s130202475

**Published:** 2013-02-18

**Authors:** Marc Currie, Pouya Dianat, Anna Persano, Maria Concetta Martucci, Fabio Quaranta, Adriano Cola, Bahram Nabet

**Affiliations:** 1 Optical Sciences Division, U.S. Naval Research Laboratory, Washington, DC 20375, USA; 2 Electrical and Computer Engineering Department, Drexel University, Philadelphia, PA 19104, USA; E-Mails: pouya.dianat@gmail.com (P.D.); bahram.nabet@drexel.edu (B.N.); 3 IMM-CNR, Unit of Lecce, Via Monteroni, I-73100 Lecce, Italy; E-Mails: anna.persano@le.imm.cnr.it (A.P.); concetta.martucci@le.imm.cnr.it (M.C.M.); fabio.quaranta@le.imm.cnr.it (F.Q.); adriano.cola@le.imm.cnr.it (A.C.)

**Keywords:** photodetector, photodiode, GaAs, low-temperature grown GaAs, electro-optic sampling, ultrafast detector, heterojunction, Schottky contact

## Abstract

Low temperature growth of GaAs (LT-GaAs) near 200 °C results in a recombination lifetime of nearly 1 ps, compared with approximately 1 ns for regular temperature ∼600 °C grown GaAs (RT-GaAs), making it suitable for ultra high speed detection applications. However, LT-GaAs detectors usually suffer from low responsivity due to low carrier mobility. Here we report electro-optic sampling time response measurements of a detector that employs an AlGaAs heterojunction, a thin layer of LT-GaAs, a channel of RT-GaAs, and a vertical electric field that together facilitate collection of optically generated electrons while suppressing collection of lower mobility holes. Consequently, these devices have detection efficiency near that of RT-GaAs yet provide pulse widths nearly an order of magnitude faster—∼6 ps for a cathode-anode separation of 1.3 *μ*m and ∼12 ps for distances more than 3 *μ*m.

## 1. Introduction

GaAs grown by molecular beam epitaxy (MBE) at low, near 200 °C, temperature (LT-GaAs) has become the material of choice for ultra high speed (such as THz) detection [1,2], due to its very short electron lifetime of around 1 ps and hole lifetime of around 12 ps [3]. By contrast, regular temperature GaAs (RT-GaAs) is grown at around 600 °C and has carrier lifetime of approximately 1 ns. This short lifetime requires that optically generated carriers be collected quickly to achieve a device with high-responsivity. However, LT-GaAs can have very low electron and hole mobilities [4,5], resulting in low photocurrent and necessitating very short, around 100 nm, cathode-anode separation gaps [6–9]. These short gaps increase capacitance. Hence, in order to avoid RC time constant limitations, devices must have small area, further reducing responsivity. We have previously designed a novel structure that circumvents this limitation [10] and have reported a responsivity of 0.15 A/W that was unprecedented for LT-GaAs, with a 12 ps pulse width, full width half max (FWHM), measured optoelectronically (OE). However, despite this high detection speed considering the device dimensions, it was ultimately limited by our instrumentation.

Here, we present electro-optic sampling (EOS) time response data with ∼1 ps resolution. The device achieves high responsivity near that of RT-GaAs while maintaining the high response speed of LT-GaAs. This is done by utilizing: (1) an AlGaAs heterojunction with a thin channel of RT-GaAs constructed for better collection efficiency of carriers, (2) a thin (85 nm) LT-GaAs layer below this RT-GaAs channel that maintains high speed by capturing slow carriers, and (3) a vertical electric field transverse to cathode–anode direction that guides electrons to the high-speed long-lifetime channel and intercepts the low speed holes. These features result in a photodetector with a dark current in tens of picoamps (hence large signal-to-noise ratio) that demonstrates a high-speed response with a 6.3 ps pulse width (measured by EOS, which is nearly half of what OE measurements have shown [10]) and with a responsivity that is comparable to RT-GaAs. We model the electric field within the structures to gain better insight into the performance, especially in balancing the vertical and horizontal electric fields. The EOS results also demonstrate trade-offs in the effect of the vertical built-in field versus the horizontal field that is due to the Schottky contacts and the external bias in the devices, confirming our device simulations and the physical basis for the enhanced performance of this detector.

## 2. Experimental Section

**Figure 1.**
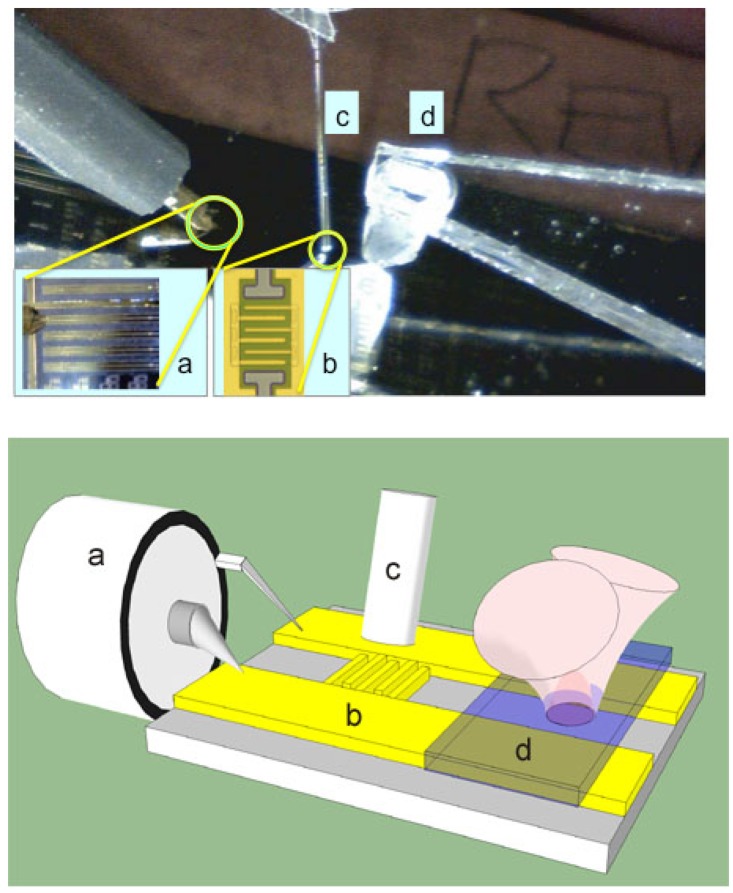
Image (top) and sketch (bottom) of the electro-optic sampling test setup highlight the (a) RF probe contacting the transmission line, (b) MSM photodetector, (c) optical fiber and (d) electro-optic crystal. The RF probe (a) is at one end of a transmission line, in the middle of which the interdigitated MSM device (b) is located. An inset shows an image of the fabricated device (b). The pump beam is delivered by an optical fiber (c) and the probe beam samples the propagating transient through the electro optic crystal (d).

Figure 1 shows the EOS measurement setup. The device under test is an interdigitated metal-semiconductor-metal (MSM) detector with two Schottky contacts for cathode and anode, which sits on a co-planar transmission line (TL), shown in inset as Figure 1(a), required for EOS measurement. Devices with finger spacing ranging from 1.1 to 8.7 *μ*m (and finger width in 1–2 *μ*m range) were fabricated in order to study the effect of transit distance. A 40 × 40 *μ*m^2^ device area results in measured capacitance of <40 fF, ensuring that none of the devices are RC time constant limited.

**Figure 2.**
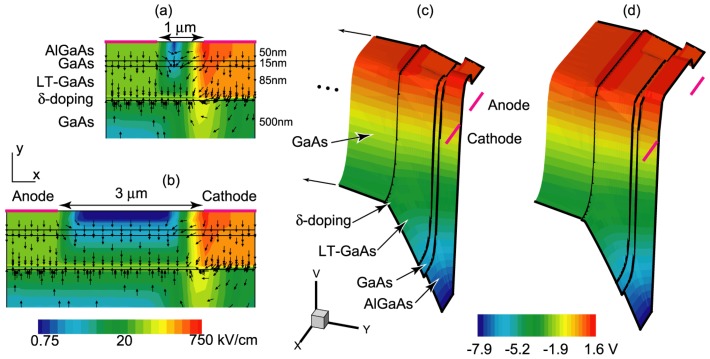
Electric field distribution in devices with 1-*μ*m (**a**) and 3-*μ*m (**b**) electrode spacings under 9 V applied bias. The layer structure is also shown in the vertical axis, and has a different scale from horizontal axis. Vectors show the calculated direction of the electric field. The electrostatic potential diagrams for the 1-*μ*m (**c**) and 3-*μ*m (**d**) detail the effects of the Schottky contact, bias, and the δ-doping profile.

The MBE growth was performed on a semi-insulating (100) GaAs substrate, starting with a buffer layer, followed by a 500 nm thick layer of unintentionally doped GaAs and a p-type (C-dopant) delta doping layer. An 85 nm LT-GaAs active layer was then grown at 300 °C, followed by a 15 nm undoped GaAs layer grown at the regular growth temperature of 600 °C, producing a channel with high mobility and long lifetime, hence momentum relaxation distance, for carrier transport. Growth of a 50 nm AlGaAs layer formed a heterojunction with this RT-GaAs channel. Figure 2(a,b) shows the layer structure and the calculated electric field distribution in devices with 1- and 3-*μ*m electrode spacings under 9 V applied bias, respectively. Horizontal and vertical axes have different scales. Nevertheless, the electric field vectors are primarily vertical, and are in the direction of growth. The electrostatic potential is shown on the right side in Figure 2(c,d), for the 1- and 3-*μ*m spaced devices, respectively, using Synopsys Sentaurus TCAD device simulator, and modeling the LT-GaAs with trap density, type, and carrier mobility and lifetime values given in [4,5]; they also show the effect of vertical (y-direction) field lines in the LT-GaAs and RT-GaAs channel regions.

**Figure 3.**
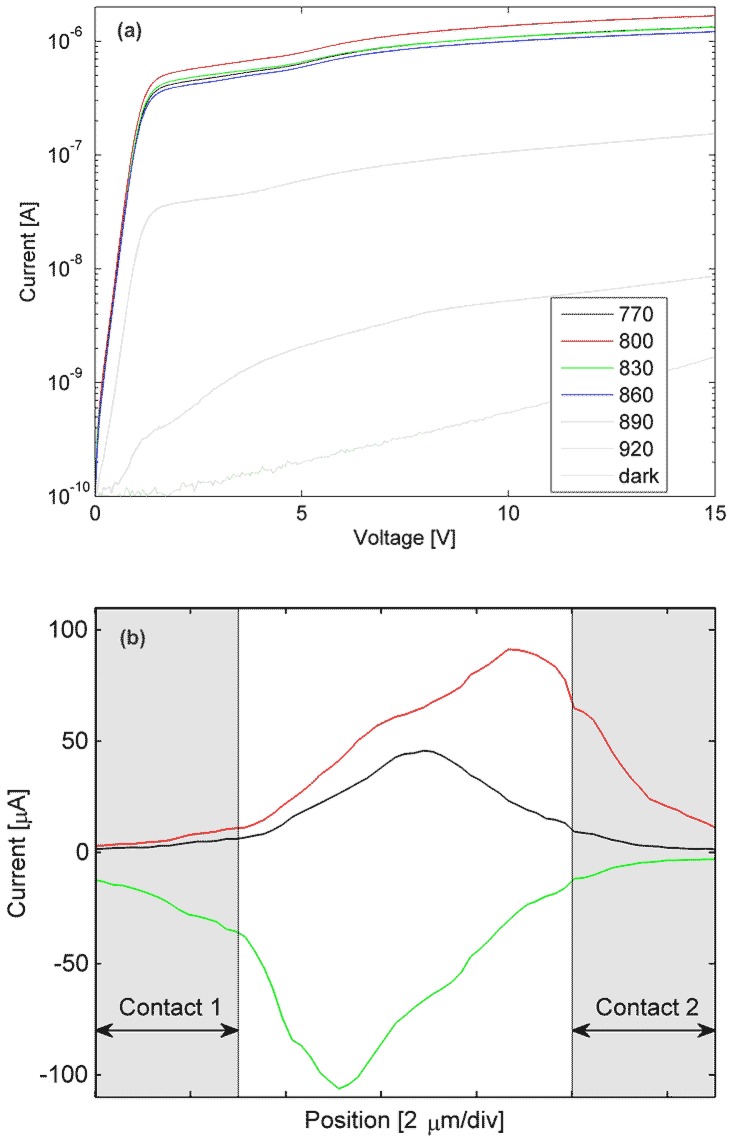
(**a**) Current-voltage curves of the 1.3 *μ*m device illuminated with 11 *μ*W average optical power at wavelengths ranging from 770–920 nm, as well ambient room lights. (**b**) Photocurrent profiles of a 1 *μ*m diameter, 200 *μ*W laser as it is translated across a 7 *μ*m electrode spacing for three applied bias voltages +5, +2, and –5 V (red, black and green traces, respectively). The gray shaded regions show the position of each electrode.

Four photodetector geometries were fabricated, which varied the separation between anode and cathode. The interdigital spacings were slightly asymmetric, producing average distances of 1.3, 3.2, 5.1 and 8.1 *μ*m, for the four devices. For the narrowest electrode gap (1.3 *μ*m), the average current–voltage measurements are shown in Figure 3(a) at an average optical power of 11 *μ*W provided by a Ti:Sapphire source for the wavelength range of 770 to 920 nm. The data in Figure 3(a) show a similar response for 770–860 nm light with a peak current response at 800 nm. At 890 nm and 920 nm, the wavelengths are near or below the band gap of GaAs so the photo-excited current decreases dramatically, although since we excite with short optical pulses their spectra even at 920 nm contain energy above the GaAs band edge. Using optical reflectance measurements (not shown), the GaAs and AlGaAs band edges are observed at 890 nm and 680 nm, respectively, as expected [11]. The samples exhibit a low dark currents of <0.5 nA at 10 V resulting in a high signal-to-noise ratio (>65 dB, as shown in Figure 3(a) even at the low excitation power of 11 *μ*W.

Spatial mapping of photocurrent within the electrode gap spacing is performed by scanning a ∼1 *μ*m diameter laser spot across a 7 *μ*m electrode spacing, for various bias voltages. Figure 3(b) shows that when a 200 *μ*W *cw* laser (780-nm wavelength) excites our sample, a peak current response is near the center of the gap for small applied bias (∼2 V), and for large applied bias (+5 or –5 V) the peak response is near the anode. This may be explained by considering the electric field distribution depicted in Figure 2. At large biases, the horizontal field at the cathode may reach the anode—a condition known as flat-band [12]. In that case, electrons generated in the proximity of the anode within a collection distance, which in this device is set by the LT-GaAs layer and facilitated by the vertical field, are the main sources of current flow. However, when the beam is near the cathode, optically generated holes are the main components of current [13], whose collection is impeded by the vertical field that pushes them through the LT-GaAs into the substrate. As a result, when the beam is near the anode, much higher current flows. If the bias polarity changes, the other contact becomes the anode, and as expected, maximum current is observed in its vicinity. For small biases, a rather symmetric energy band diagram produces maximum photocurrent near the center of the gap.

**Figure 4.**
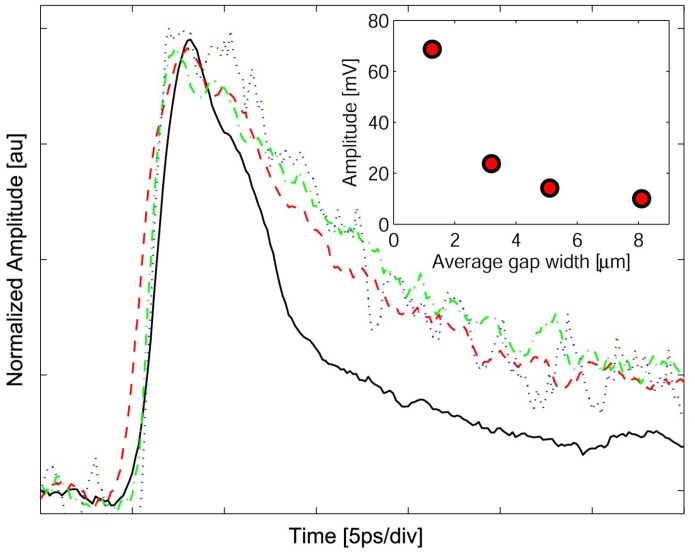
Time response measured by electro-optic sampling of devices with 1.3, 3.2, 5.1, and 8.1 *μ*m separations between cathode and anode (black, red, green and blue traces, respectively). A 6.3 ps FWHM pulse width and a 0.15 A/W responsivity is observed for the smallest device while all others separations have a ∼12 ps FWHM pulse width. Inset of the figure shows the peak response for each device (scaled to the device's active area).

Time responses were measured by integrating the device into a transmission line and testing with an EOS system. In essence, the EOS is an ultrafast sampling oscilloscope that uses 150 fs laser pulses to excite optoelectronic transients, and then measures the electronic response by probing the refractive index change of an electro-optic crystal placed on top of the device and/or transmission line. In our setup the laser is split into two paths: one coupled into a fiber (Figure 1(c)) to excite the device (Figure 1(b)), and another path that passes through a LiTaO_3_ crystal (Figure 1(d)) to sample the electric field of the propagating response. By varying the optical path of the sampling beam, the temporal response of the device is observed with a time-resolution limited by the laser pulse width and the response of the electro-optic crystal. In this experiment we used ∼150 fs pulses from a Ti:sapphire laser with a center wavelength of 830 nm. Our EOS system has a 450 fs response time, which is primarily limited by the optical pulse widths: a 400 fs switching pulse at the fiber output and a 150 fs sampling pulse within the LiTaO_3_ crystal. The MSM photodetector's electronic response is coupled to a coplanar strip transmission line that is contacted with microwave probes (Figure 1(a)) for DC bias and <50 GHz measurements. The data shown in Figure 4 are for 1.3, 3.2, 5.1, and 8.1 *μ*m gaps between cathode and anode. For all of the MSM devices, the rise time is approximately 2 ps; the fall times are similar, except for the device with the 1.3 *μ*m electrode separation. This provides a pulse width of 6.3 ps FWHM for the 1.3 *μ*m structure and a 12 ps FWHM pulse width for the wider spacings that are, interestingly, independent of the gap distance.

For comparison, similar devices were fabricated on identical structures where the LT-GaAs was replaced with RT-GaAs (otherwise all parameters were the same, including the p-delta doping layer). They showed time responses ranging from 16 to 100 ps FWHM depending on the electrode spacing.

## 3. Results and Discussion

Several factors account for the high speed and high collection efficiency of this device. The first is the vertical electric field produced by the GaAs/AlGaAs hetero-interface [14] and increased by the p-type delta-doping, which forces the optically generated electron up towards the interface, and the holes down through the LT region. The second is the existence of a 15 nm RT-GaAs channel that allows long recombination lifetimes and high mobility transport while also providing a good hetero-interface with AlGaAs unaffected by the deep defects of LT-GaAs. The third is the 85 nm thick layer of LT-GaAs that is sandwiched between the channel and the p-delta doping layer. As a result, the transport of the optically generated electron and hole pairs (EHP) in various depths of the device is affected and may be examined as follows.

The EHPs that are generated within the short 15 nm channel will be collected within their nanosecond lifetime at anode and cathode, respectively, given the usual high mobility of RT-GaAs. In LT-GaAs, however, free electrons (holes) are expected to have a lifetime of about 500 fs (12 ps), and mobility of ∼500 (∼90) cm^2^/V·s [5]. The electric field profile of Figure 2(a,b) shows that the electrons generated in the 85 nm LT-GaAs would primarily travel up to the RT-GaAs channel and can then be collected under the high field of the anode. The holes generated in the same region will have ∼12 ps to be collected; however, the vertical field under the cathode traps the low mobility holes in the LT-GaAs. The EHPs generated deeper into the GaAs substrate would also follow the field lines with electrons having to transit primarily the 85 nm thickness of the LT-GaAs, not the several micron distance between cathode and anode. The slow holes, on the other hand, are prevented from reaching anode due to the LT layer and the vertical electric field. The interception of the slow carriers may provide an explanation for the observed behavior in Figure 4 that the time response of 3.2, 5.1, and 8.1 *μ*m devices are indistinguishable and independent of the transit distance. The device with the smallest gap, however, has a shorter response time, and therefore an additional dynamic mechanism is occurring.

The balance between the horizontal component of the electric field (*i.e.*, between the electrodes) and the vertical component of the electric field (*i.e.*, from the electrode into the substrate) determines the carrier propagation direction and hence the device response. Examining the device structure, we calculate >0.2 eV band bending across the LT-GaAs due to p-delta doping, providing a vertical field of >20 kV/cm, whereas the horizontal electric field changes from nearly 160 kV/cm for the narrow spaced devices to < 10 kV/cm for the wider spaced devices for the applied bias of 9 V. Hence, for larger devices, following the field lines, carriers would need to traverse diagonally across the LT-GaAs region before reaching their respective contacts, which will increase the chance of trapping and recombination.

Another notable feature is the decrease in pulse amplitude with increasing gap width (peak pulse voltages of 35, 15, 10 and 8 mV, respectively). The smallest electrode gap width provides the shortest pulse but has the highest pulse amplitude. The three larger electrode widths (2.5, 4 and 6 times that of the narrow gap) have a longer, yet similar, time response but decrease in pulse amplitude from 15 to 8 mV. The inset in Figure 4 shows the decrease in pulse amplitude with increasing electrode width, scaled to the fraction of the device's active area to that of the total area; since a larger electrode gap has a larger active area (less Au electrode area). The amplitude response can be explained by the trapping in the LT-GaAs layer, which effectively acts as a gate for carrier collection, e.g., those carriers that cannot traverse the layer before the gate closes are not collected, thus rewarding the closely spaced electrodes. In addition, Figure 2(a–d) shows that, as expected, the bias is dropped across the cathode; hence, the field profile underneath, and in its vicinity, do not depend on the distance to anode. As a result, collection of the holes at cathode becomes independent of anode–cathode spacings as long as the band bending does not reach through to anode. By the same token, electron transit distance to anode is limited by the LT-GaAs lifetime and will not depend on gap spacings larger than reach-through.

## 4. Conclusions

By landscaping an electric field to move carriers a short distance to a high-speed channel while intercepting and trapping slow carriers in the LT-GaAs layer, we have created an efficient and high-speed photodetector, whose responsivity is much higher than previously reported LT-GaAs devices, and compares favorably with other high speed devices [10]. We used electro-optic sampling to detect the electric pulse a distance of 250 *μ*m away from the MSM device on a coplanar strip transmission line, thus the pulse dispersion due to this propagation distance can be observed by the relatively slow 2 ps rise time. However, even without compensating for the transmission line dispersion, we measured 12 ps FWHM pulse widths for our wide (3 to 8 *μ*m) electrode gaps, while our 1.3 *μ*m device produced a 6.3 ps FWHM response. Intrinsic device response is of course even faster and may be estimated by deconvolution of the system response. Our model agrees with the experimental results and demonstrates the balance between vertical and horizontal electric fields for achieving improved performance. Thus, by employing a thin (85 nm) layer of LT-GaAs in conjunction with a much thicker (500 nm) RT-GaAs absorption region, a doping profile to create a vertical field, and an AlGaAs hetero-interface with a narrow channel of RT-GaAs for efficient carrier transport, we have produced a high-speed efficient detector with a large (>65 dB) dynamic range.
